# Well-being of veterinarians in rural and urban areas

**DOI:** 10.3389/fvets.2023.1276229

**Published:** 2023-11-16

**Authors:** Cecile Gonschor, Robert Pohl, Ulrike Woitha, Beatrice Thielmann, Irina Böckelmann

**Affiliations:** Department of Occupational Medicine, Medical Faculty, Otto-von-Guericke University Magdeburg, Magdeburg, Germany

**Keywords:** well-being, veterinarians, prevention, work conditions, occupational health

## Abstract

**Introduction:**

The field of veterinary medicine is characterized by a variety of challenging working conditions. The alarmingly low mental well-being of veterinarians has been examined from various perspectives. However, the influence of work location on the well-being of veterinary professionals has scarcely been investigated. The aim of the study was therefore to analyze the well-being of German veterinarians and to determine whether there is a correlation between well-being and work location.

**Methods:**

As part of a cross-sectional study, 999 veterinary professionals answered questions regarding their work location (self-designed questions) and well-being (WHO-Five Well-being Index, WHO-5). A differentiation was made according to work location: urban cities (population > 100,000), medium/small cities (population < 100,000 inhabitants), and rural areas.

**Results:**

Overall, the surveyed veterinarians had low well-being (ranging from 56.8% in rural areas to 61.3% in medium/small towns). The results of the general linear model indicated a significant difference in the WHO total score among veterinarians from different work locations (*p* < 0.001). However, when sex, age, type of employment, and field of specialization were included in the analysis, no significant between-subject effects were found.

**Conclusion:**

According to the results, work location does not seem to have a significant influence on the well-being of veterinarians and therefore may be of lower priority in the development and implementation of interventions. However, further investigation of work-related predictors of the mental health of veterinarians is recommended, as the results indicate a low well-being among these professionals.

## 1. Introduction

The field of veterinary practice and its impact on mental well-being have the subject of an increasing number of international studies. Numerous results indicate high prevalences of various risk factors for mental disorders among individuals in this profession (1). Veterinarians show higher levels of stress (2–5), burnout (2, 6), depression (2, 3, 7), and suicidality (2, 3, 5, 7–9) than the general population and other occupational groups. Risk factors associated with poor mental health and high suicide rates among veterinarians include constant exposure to difficult scenarios such as interpersonal conflict, the performance of euthanasia and easy access to lethal suicide drugs such as opioids and anesthetics, highlighting the need to better understand predisposing factors and improve mental health in this professional environment, as well as introduce primary mental health care interventions (10). In an American study, Volk et al. (11) reported a lower level of well-being among veterinarians compared to the general population. The association between poor working conditions and low well-being is also reflected in the literature (6, 12). According to the definition of the World Health Organization (WHO), well-being is a general term that encompasses various areas of human life, including physical, mental, and social aspects (13). The field of veterinary medicine in various countries, including Germany, is characterized by long working hours, excessive duties after work, low remuneration, unexpected outcomes of clinical cases, conflicts with clients, the performance of euthanasia, and the restriction of treatments due to clients’ financial situations (12, 14). However, working conditions and resources are not the only variables influencing well-being. Sex, marital status, age, and field of specialization have been investigated as predictors of well-being. The components of well-being and job satisfaction have a major impact on health (13). Other work-related stress factors affecting veterinarians also appear to be related to fair pay. A sample of German veterinarians showed that approximately one-third (29.4%) do not consider their wages to be performance-related or fair (15). The performance of euthanasia (16) or the permanent expectations of patient owners (14) also has a negative effect on psychological stress and likewise on well-being. Compassion satisfaction, burnout risk and traumatic stress may also be positively influenced by income or work experiences among veterinarians (17).

Hatch et al. (18) reported a significant difference in the prevalence of burnout and depression among veterinarians working in different locations in Australia. Accordingly, veterinarians in large and small to medium-sized cities were more affected by depression and burnout than those working in rural areas. In Germany, many veterinarians nevertheless choose to run small animal practices in urban areas, which, at the same time, leads to a shortage of specialists for large animals in rural areas (19). The anticipated shortage of farm animal veterinarians is perceived by the British Cattle Veterinary Association (2019) as one of the greatest challenges facing farm animal medicine. The reasons for this include not only the decrease in the number of farms where farm animals are kept but also the fact that regulated working hours and a better work-life balance often lead to a focus on specialization in the treatment of small animals, which are increasingly kept in urban areas (20). However, practice ownership has a positive impact on burnout risk among private practice veterinarians but does not affect traumatic stress (21). It is currently unclear to what extent the lack of staff in rural areas and the simultaneously high workload in urban areas affect the health of veterinarians in Germany.

Based on existing studies, it is clear that there is a need for health-promoting and preventive interventions in the field of veterinary medicine, particularly regarding mental health. The occupational medical care provided by company doctors places great emphasis on the assessment of psychological stressors and the early detection and prevention of psychological symptoms through counseling with employees. To ensure the success of these interventions, it is essential to consider the specific needs of the target group. Therefore, further studies about the well-being of veterinarians are necessary to integrate as many predictors as possible regarding the development of health promotion and prevention measures. The aim of the observational cross-sectional study presented here was to assess the well-being of German veterinarians and to analyze whether there is a correlation between the well-being of veterinarians and their work location. This will provide an initial assessment of the relevance of work location in designing interventions. The present study examined the extent to which the results of previous studies are applicable to Germany. It was hypothesized that the well-being of veterinarians would differ among different types of work locations, such as urban areas (population > 100,000 inhabitants), small to medium-sized towns (population < 100,000 inhabitants), and rural areas.

## 2. Methods

The present study was part of the ongoing nationwide survey “Causes and consequences of psychological stress in the working life and emergency services of veterinary professionals in the Federal Republic of Germany.” The study consists of an observational cross-sectional design with selected standardized instruments for evaluating stress, strain, health status, and well-being, in addition to occupational medicine-psychological questionnaires, completed through an online survey (SoSiSurvey). Considering the presented hypothesis, the data from the WHO-5 well-being questionnaire were evaluated (22). Since the sample consisted exclusively of veterinarians (employed in Germany), the entire quantitative survey consisted of items in German.

Furthermore, specific questions on sociodemographic data, job characteristics, and organizational conditions were developed in collaboration with the Veterinary Chamber of Saxony-Anhalt. Accordingly, specific comparisons between certain target groups, such as comparisons based on the work territory (urban area, small town, rural area), were possible. A detailed description of the study and the methodological approach is described in the published study protocol (23).

Recruitment was undertaken using advertising opportunities through the German Veterinary Association, the state veterinary associations and through additional information in the journal Deutsches Tierärzteblatt (issue 09/2021), as well as via social media (Facebook, Instagram). In addition to a link to the study, a QR code was generated so that subjects who were willing to participate could access the online survey directly. The study included veterinarians who worked in a wide variety of fields in the Federal Republic of Germany (small animals, horses, farm animals, laboratory field and public authority) and had been working in their fields for at least 1 year. Overall, 1,170 veterinarians voluntarily participated in the online survey that took place from July 1, 2021, to January 1, 2023. The sample consisted of veterinarians from different fields of work (self-employed, employed, government employees, assistant doctors, etc.) and different work locations (urban cities with a population higher than 100,000 (“capital cities”), medium/small towns with a population lower than 100,000 inhabitants, and rural areas). After eliminating participants with missing data, those who had completed the WHO-5 and indicated their work location were included in the analysis (*n* = 999).

Well-being was assessed using the five items of the standardized and validated WHO-5 questionnaire (22). The scale uses a six-point Likert-type scale ranging from 0 = ‘at no time’ to 5 = ‘all of the time’. The reliability of the WHO-5 was assessed by analyzing the internal consistency (Cronbach’s *α* = 0.92) as well as the test semi reliability according to Guttmann (0.87) and was rated as good. The evaluation was performed according to the evaluation method specified by the WHO (22). The total score value (0–25) was used as an indicator of overall well-being. A raw score value below 13 indicated poor or low well-being, while scores of 13 or higher implied good or high well-being (22). Information on the work location was obtained through a self-designed question in the section on occupational information. Overall, the WHO-5 had very good psychometric performance. Its indices had satisfactory psychometric properties, both in terms of reliability, construct validity and the utilization of the scale range (24).

Based on the division of the sample into three groups (large cities, medium/small towns, and rural areas) and the nonmetric measurement level, the nonparametric Kruskal–Wallis test and *post hoc* Bonferroni correction were performed to determine differences among the three work location groups. The effect size of the corrected model in the general linear model was represented by the partial eta-squared. The significance level was set at 5%. Statistical analysis was performed using SPSS version 28 (IBM, Armonk, NY, USA).

## 3. Results

The average age of the present sample was 41.7 ± 10.19 years. The youngest participant at the time of the survey was 23 years old, and the oldest was 79 years old (Table 1). The majority (64.9%) of the participating veterinarians were female. Regarding work location, an equal distribution was observed. Most surveyed veterinarians worked in rural areas (39.1%), followed by medium/small towns (33.6%) and large cities (27.2%). On average, the work experience of the sample was 14.3 ± 9.95 years (Table 1).

**Table 1:** Descriptive statistic of the variables age and professional experience categorized by work location.

Variables	Total	Large cities (population: > 100,000)	Medium/small cities (population: < 100,000)	Rural area	p_Kruskal-Wallis-test_
	Mean ± Standard deviation				
Age [in years]	41.7 ± 10.20	40.81 ± 10.213	41.82 ± 9.887	42.31 ± 10.431	0.190
Professional experience [in years]	14.3 ± 9.95	13.23 ± 9.749	14.50 ± 9.621	14.76 ± 10.336	0.116

The well-being of the majority of the sample (59.4%) was classified as low (raw score < 13). The investigated sample showed a low level of well-being across all work locations, with a raw score of 11.1 ± 5.30 points (Table 2). There was no significance in the Kruskal–Wallis analysis, as shown in Tables 1 2; therefore, the *post hoc* test was not performed.

**Table 2:** Descriptive statistic of the variables well-being categorized by work location.

Variables	Total	Large cities (Population: > 100,000)	Medium/small cities (Population: < 100,000)	Rural area	p_Kruskal-Wallis-test_
	Mean ± Standard deviation Median (Min – Max) 95% confidence interval				
Well-being	11.1 ± 5.30	10.71 ± 5.276	10.90 ± 5.273	11.43 ± 5.339	0.250
	(0–25)	(0–22)	(0–23)	(0–25)	
		[10.08 – 11.34]	[10.34 – 11.47]	[10.90 – 11.97]	

Since the distribution of sex within the workplace groups was rather proportional (Pearson’s chi-squared test, *p* = 0.455), further statistical analyses for work location groups were conducted for both sexes combined (Table 3). To test for a significant relationship of the categorical variables (of the variables sex, department and type of employment categorized by work location), the chi-square test (χ^2^) was also used (Table 3).

**Table 3:** Descriptive statistic of the variables gender, department and type of emplyment categorized by work location.

		Large cities (Population: > 100,000)	Medium/small cities (Population: <100,000)	Rural area	Total *n* = 999	p_*χ*2 nach Pearson_
Gender						
Male	Quantity	104	114	133	351	0.455
	% of gender	29.6%	32.5%	37.9%	100.0%	
	% of total	10.4%	11.4%	13.3%	35.1%	
Female	Quantity	168	222	258	648	
	% of gender	25.9%	34.3%	39.8%	100.0%	
	% of total	16.8%	22.2%	25.8%	64.9%	
Department						
Small animals	Quantity	196	241	113	550	<0.001
	% of department	35.6%	43.8%	20.5%	100%	
Large animals (livestock and horses)	Quantity	15	23	134	172	
	% of department	8.7%	13.4%	77.9%	100%	
Small and large animals	Quantity	4	37	108	149	
	% of department	2.7%	24.8%	72.5%	100%	
Laboratory	Quantity	23	9	2	34	
	% of department	67.6%	26.5%	5.9%	100%	
Public authority	Quantity	33	26	32	91	
	% of department	36.3%	28.6%	35.2%	100%	
Other	Quantity	1	0	2	3	
	% of department	33.3%	0%	66.7%	100%	
Type of employment						
Self-employed/practitioner	Quantity	79	139	185	403	<0.001
	% of employment	19.6%	34.5%	45.9%	100%	
Public sector employee	Quantity	47	28	26	101	
	% of employment	46.6%	27.7%	25.7%	100%	
Civil servant	Quantity	15	7	10	32	
	% of employment	46.9%	21.9%	31.3%	100%	
Trainee/assistant doctor	Quantity	69	89	96	254	
	% of employment	27.2%	35%	37.8%	100%	
Doctoral student	Quantity	6	2	0	8	
	% of employment	75%	25%	0	100%	
Employed in a practice/clinic	Quantity	45	52	56	153	
	% of employment	29.4%	34%	36.6%	100%	
Other activity	Quantity	5	7	6	18	
	% of employment	27.8%	38.9%	33.3%	100%	
Private sector	Quantity	6	11	12	29	
	% of employment	20.7%	37.9%	41.4%	100%	
Without professional practice	Quantity	0	1	0	1	
	% of employment	0%	100%	0%	100%	
Total	Quantity	272	336	391	999	
	% of total	27.2%	33.6%	39.1%	100%	

Table 4 presents the distribution of the work location groups with different levels of well-being. Overall, a comparable ratio between low and high well-being was found across all work locations (Pearson’s chi-square test, *p* = 0.407). Veterinarians with low well-being predominated those with high well-being in large citys and medium/small cities.

**Table 4:** Low and high well-being by work location.

Variables	Low well-being *n* (%)	High well-being *n* (%)	p_*χ*2 nach Pearson_
Large cities (Population: > 100,000)	165 (27.8%)	107 (26.4%)	0.407
Medium/small cities (Population: < 100,000)	206 (34.7%)	130 (32.0%)	
Rural area	222 (37.4%)	169 (41.6%)	
Total	593	406	

Low and high well-being by work location (Pearson’s *χ*^2^-test: *p* > 0.05). Well-being measured with the WHO-5 questionnaire: a raw score value below 13 indicates poor or low well-being, while scores of 13 or higher imply good or high well-being.

The results of the general linear model (Table 5) revealed a significant difference in the WHO total score among veterinarians from different sized work locations in the corrected model (*p* < 0.001). However, when sex, age, employment type, and work location were included in the analysis, no significant between-subject effects were found between well-being (dependent variable) and the listed independent variables. The department was not taken into account in this model due to its strong correlation with both workplace and well-being. The comparison of the WHO total and the work location did not result in any significant statement (regardless of whether considered alone or in combination with the other possible influencing variables). The assumptions of homogeneity of variances were found to be satisfied, as assessed by Levene’s test (*p* = 0.802). The Heteroskedastizität Test (modified Breusch–Pagan-Test) was not significant (*p* = 0.074). The homogeneity of regression slopes were not violated with regard to the dependent variable, as the interaction terms were not statistically significant (*p* = 0.249).

**Table 5:** Results of the general linear model for the variable well-being (AV) in workplace groups with the consideration of covariates and interaction terms.

	Well-being (WHO-5)	
Corrected model	Median of the squares	84.376
	F	3.077
	*p*	<0.001
	η^2^	0.036
Gender	Median of the squares	56.755
	F	2.070
	*p*	0.151
	η^2^	0.002
Age	Median of the squares	198.399
	F	7.235
	*p*	0.007
	*η* ^2^	0.007
Type of employment	Median of the squares	28.483
	F	1.039
	*p*	0.402
	*η* ^2^	0.007
Work location	Median of the squares	42.443
	F	1.548
	*p*	0.213
	η^2^	0.003

R-squared = 0.024.

We chose logistic regression (including Wald test) with all considered coefficients (explanatory variables). Estimation stopped at iteration number 4 because the parameter estimates changed by less than 0.001.

The department was not taken into account in this model due to its strong correlation with both workplace and well-being.

*F* = between-group variance.

η^2^ = measure of effect size (0 = no effect; 1 = the independent variable explains all the variance of the dependent variable).

*p* = level of significance (*p* > 0.05 not significant).

Type of employment refers to employment relationship, such as: Self-employed/practitioner, public sector employee, civil servant, trainee/assistant doctor, doctoral student, other.

Department refers to the work environment, such as: small animals, large animals (livestock and horses), laboratory, public authority, other.

Work location refers to large cities, medium/small cities and rural area.

## 4. Discussion

Currently, there is a limited amount of research examining the relationship between the well-being of veterinarians and their place of work. This study provides initial insight into a potential association between well-being, as measured by the WHO-5, and the work location of veterinary practitioners. The sample was divided into three groups based on the practitioners’ place of work, i.e., large urban areas, small to medium-sized towns, and rural areas. The findings suggest that the well-being of the majority of participating veterinarians is low, regardless of their work location. This result is reflected both independently and in relation to the place of work. A significant difference in the WHO total score was observed among the different work locations in the adjusted model (*p* < 0.001). There were no significant intersubject effects between well-being and sex, age, specialty, or employment type.

The impact of work and education on well-being is generally well researched and clear. A 2017 World Happiness Report study showed clear differences in life satisfaction and emotional quality between employed and unemployed people (25). Furthermore, research has shown that well-being is strongly dependent on job characteristics. Meta-analytical studies have confirmed that working conditions such as rewards, communication, leadership quality and job security have significant effects on employee well-being (25). In this respect, well-being is dependent on many occupational characteristics. However, work location has hardly been studied thus far, although the work location of veterinary professionals could definitely play a role.

Various studies have indicated that work-related stress is a major risk factor for poor well-being (26) and is associated with burnout (27) and various cardiovascular diseases (28). Hatch et al. (18) reported significant differences in burnout and depression prevalence among veterinarians with different work locations. Veterinarians working in urban areas and small to medium-sized towns (“rural city or town”) were found to have a higher risk of burnout and depression than their colleagues working in rural areas. It should be noted that the WHO-5 has rarely been utilized in the veterinary field to assess general well-being in target groups. Instead, its focus has been on identifying specific health issues in veterinary medicine, such as depression or suicidality. As per the literature, the use of the WHO-5 is predominantly recommended in conjunction with a preexisting diagnosis (e.g., diabetes) or certain sociodemographic characteristics (e.g., students, pregnant women, older adults). However, the differences in well-being by work location have yet to be extensively investigated using the WHO-5. The study by Heringshausen et al. (29), examining the well-being of emergency responders in Germany according to their work location, demonstrated a significant difference in WHO-5 scores (*p* ≤ 0.01). Emergency responders stationed in small towns (population of 5,000-20,000) had notably higher well-being scores than their counterparts stationed in large cities (population > 100,000). Nonetheless, based on the present study’s findings and the limited research available, work location cannot be considered a reliable predictor for well-being in veterinarians in Germany.

In their study, Macía et al. (30) showed that more than half (53.2%) of a veterinary sample from Spain had average mental well-being, while 37.9% reported high mental well-being, and only 9.0% had low scores (30). These results are more positive compared to those of the present study (59.4% with low well-being), although it should be noted that both studies used different instruments (Macía et al. used the Warwick-Edinburgh Mental Wellbeing Scale).

Contrary to research regarding work location and well-being, various studies investigating different aspects of the well-being of veterinarians exist. These studies have identified difficulties with mental health and mental illnesses, particularly depression (2, 3, 12), increased stress (31), and sleep disorders (12), which have been directly associated with work-related factors. Furthermore, the previously mentioned increased incidence of suicide among veterinarians must be acknowledged as a clear warning sign (3, 7). Overall, a lower level of well-being among veterinarians compared to other occupational groups can be observed. Reis et al. (32) investigated the well-being of 771 psychotherapists using the WHO-5 and found that only 237 (30.7%) had a raw score < 13, indicating a “low” level of well-being. In the nonmedical sector, Dadaczynski and Paulus (33) found that only 36% of the 4,326 school principals in Germany had low well-being scores according to the WHO-5. Notably, occupational groups with greater comparability exhibit similar levels of well-being, with 56% of physicians and 52% of nursing staff (*n* = 389) having critical values regarding well-being (34). In the present study, 59.4% of the veterinarians demonstrated low levels of well-being. Several limitations should be considered in the study described here and the subsequent analyses. First, it should be noted that the study was based on cross-sectional data, and therefore, no definitive statements regarding causality can be made.

Furthermore, the survey was conducted online, which may have resulted in selection bias among the participants. However, it should be mentioned that this potential bias was partly counteracted by the dispersion of the survey link through the respective national veterinary medical associations, as well as through the German Veterinary Journal (the journal of the German Veterinary Medical Association). Additionally, the data are subject to potential sources of bias stemming from subjective self-assessments, which can include errors in responses or bias due to social desirability.

Ultimately, the representativeness of the study needs to be considered on a national and international level. Due to the sample size (*n* = 999) and sex distribution, there is a certain degree of representativeness to the population of veterinary practitioners in Germany compared to smaller samples. According to statistics from the Federal Chamber of Veterinarians (2022), there were a total of 32,930 veterinarians working in Germany in 2021 (20). Both the population of actively practicing veterinarians in Germany and the sample in this study exhibit a comparable sex ratio. Within the framework of the online survey, the responses of 648 (64.9%) female and 351 (35.1%) male veterinary practitioners were recorded. The population in Germany in 2021 consisted of 22,689 female (68.9%) and 10,241 male (31.1%) veterinary professionals (20).

On an international level, representativeness may vary due to differences in working conditions. However, both well-being and work location are natural constructs that can be applicable in various working conditions. It is important to consider the country-specific conditions and individual determinants resulting from these conditions.

Thus, it can be argued that well-being can be influenced by a variety of determinants. The WHO-5 measures overall well-being, which is roughly divided into two categories: “low” and “high.” Therefore, it is possible that factors that were not further examined could have an impact on the relationship between well-being and work location or that work location could have an influence on only one specific aspect of well-being. Furthermore, there may be additional influencing factors with neutralizing effects, such as social support, work density, or economic situation, which could vary with work location.

In the study by Platt et al. (7), four of the 21 interviewed veterinarians independently suggested that the need for a healthy work-life balance in veterinary medicine should be achieved by both employees and employers. Therefore, it is important for veterinarians to develop awareness and for employers to facilitate this process. Working conditions should be made more conducive to this goal, and employees should be actively encouraged to achieve a healthy work-life balance, for example, through consultation with the company physician within the framework of occupational health care. The relevance of changing work-related stressors in addition to strengthening personal resources has already been demonstrated in various studies (12, 35).

Another suggestion made by the veterinary professionals interviewed by Platt et al. was to develop a peer-to-peer or mentoring system. Participants stated that veterinarians tend to be more reserved in actively seeking help: “Do not ask vets if they need it…they will say no because they are high achievers.” Therefore, it would be important to implement supportive measures such as those described above in the everyday work environment, with a low threshold for access. By integrating these measures as a standard aspect of veterinary medicine instead of optional services, it could prevent only severely affected individuals from seeking and receiving support. Moreover, it is possible that by normalizing these measures, potential barriers could be reduced, leading to not only an intervening but also a preventive effect.

Hoy-Gerlach et al. (36) described interesting approaches to reducing occupational stressors in animal shelter workers, some of which can also be applied to veterinarians, such as training for self-care and stress reduction or the integration of veterinary social workers to relieve the veterinary staff and to prevent emerging psychosocial problems among pet owners.

The hypothesis that the work location could have an impact on the well-being of veterinarians was thus rejected. The consideration of work location does not seem to be a crucial factor in the development and implementation of interventions. However, further investigation of work-related predictors of well-being in veterinarians is recommended, as the results still indicate a work-related reduction in well-being.

## 5. Conclusion

Consequently, interventions should be implemented not only at the individual level but also at the organizational level, involving both employees and employers. In Germany, the mandatory psychosocial risk assessment for mental health, as stipulated in §5 (1) of the Act on the Implementation of Measures of Occupational Safety and Health to Encourage Improvements in the Safety and Health Protection of Workers at Work (ArbSchG) (37), provides a fundamental basis for this. Similarly, in many EU countries, such psychological risk assessment is a legal obligation (35). The focus should be primarily on the ability of managers and employers to identify and minimize avoidable stressors. It is particularly recommended to assess job demands, job content, work organization, social relationships at work, and the work environment (37).

## Data availability statement

The original contributions presented in the study are included in the article/supplementary material, further inquiries can be directed to the corresponding author.

## Ethics statement

The study has been approved by the Otto-von-Guericke University Magdeburg ethics committee (No. 163/21). The studies were conducted in accordance with the local legislation and institutional requirements. Written informed consent for participation was not required from the participants or the participants’ legal guardians/next of kin in accordance with the national legislation and institutional requirements.

## Author contributions

CG: Visualization, Writing – original draft, Writing – review & editing. RP: Conceptualization, Investigation, Methodology, Writing – review & editing. UW: Writing – review & editing. BT: Conceptualization, Formal analysis, Methodology, Writing – review & editing. IB: Conceptualization, Data curation, Funding acquisition, Investigation, Methodology, Project administration, Resources, Software, Supervision, Validation, Writing – review & editing.
